# Size control of nanoparticles synthesized by pulsed laser ablation in liquids using donut-shaped beams

**DOI:** 10.3762/bjnano.16.31

**Published:** 2025-03-25

**Authors:** Abdel Rahman Altakroury, Oleksandr Gatsa, Farbod Riahi, Zongwen Fu, Miroslava Flimelová, Andrei Samokhvalov, Stephan Barcikowski, Carlos Doñate-Buendía, Alexander V Bulgakov, Bilal Gökce

**Affiliations:** 1 Chair of Materials Science and Additive Manufacturing, School of Mechanical Engineering and Safety Engineering, University of Wuppertal, 42119 Wuppertal, Germany; 2 HiLASE Centre, FZU - Institute of Physics of the Czech Academy of Sciences, Za Radnicí 828, 25241 Dolní Břežany, Czech Republic; 3 Technical Chemistry I and Center for Nanointegration Duisburg-Essen (CENIDE), University of Duisburg-Essen, 45141 Essen, Germany; 4 GROC·UJI, Institute of New Imaging Technologies, Universitat Jaume I, Av. de Vicent Sos Baynat, s/n, 12071 Castellón, Spain

**Keywords:** beam shaping, cavitation bubble, donut beam, gold nanoparticles, high-entropy alloy nanoparticles, nanoparticle size analysis, yttrium oxide nanoparticles

## Abstract

The potential to modify the size distribution of nanoparticles synthesized by pulsed laser ablation in liquids is demonstrated using a donut-shaped laser beam. In experiments on pulsed laser ablation in water of gold, yttrium oxide, and high-entropy alloy targets with both Gaussian and donut-shaped beams, we observed a significant reduction in particle size, narrowing of the size distribution width, and an improvement in sphericity when utilizing the donut-shaped laser beam. We performed time-resolved shadowgraph imaging of the laser-induced cavitation bubble, revealing a toroidal structure that overruns the ring-shaped ablation site, compared to the quasi-hemispherical bubble covering the ablation spot produced by the Gaussian beam. Based on this pioneering study, further investigation with higher temporal and spatial resolution are warranted.

## Introduction

The demand for nanoparticles (NPs) with defined particle sizes and narrow size distribution width is driven by the growing integration of nanomaterials into various industrial applications, such as medicine [1–3], catalysis [4–5], sensors [6–7], and additive manufacturing [8]. The performance of NPs typically depends on the size, requiring a monodisperse or at least monomodal size distribution. As an example, gold NPs with a narrow particle size distribution achieve a higher detection sensitivity in sensing applications [9]. Besides, NP size is critical for biomedical applications, where deviations from the optimum size can cause cellular damage or prevent membrane permeation for drug delivery [10]. Therefore, a fine and reproducible size control during NP synthesis is essential.

Pulsed laser ablation in liquids (PLAL) allows for the synthesis of colloidal NPs offering numerous advantages, such as being environmentally friendly, ensuring high chemical purity, and minimizing the need for precursors and stabilizers [11–12]. PLAL can be used to synthesize a wide range of metallic and non-metallic NPs, including alloys, (doped) oxides, and polymers [13–15]. PLAL allows for the generation of complex NPs with precise compositional control such as metastable binary core–shell NPs [16] and quinary Cantor high-entropy alloy NPs [17–18]. Such high-entropy nanomaterials are recently being discussed as game changers, providing disruptive design opportunities in multifunctional catalysts or magnetic materials [19].

PLAL-based NP production has significantly advanced in the last years to fulfill industrial-scale application requirements [20]. Waag et al. achieved productivities of several grams per hour for various metal NPs using a high-power, high-repetition-rate picosecond laser in combination with a fast polygon laser scanner [21]. However, narrowing the NP size distribution remains a challenge for PLAL [22]. Typically, PLAL-generated NPs exhibit broad and/or bimodal size distributions [23–24]. Extensive research has been conducted to reduce NP size dispersion by PLAL, mainly by adding surfactants [25–26], ligands [27], and low-salinity ions [28], or by choosing different liquid media [29]. Nevertheless, the addition of molecular stabilizers or the use of liquids that increase the stability reduces the versatility of the technique and limits the applicability of the produced colloidal NPs. The most elegant, surfactant-free size quenching method by micromolar anion addition only works for soft Lewis-acid nanoparticle materials, in particular, Au, Pt, and Pd, as the process is driven by anion adsorption Hofmeister effects, but not for oxide or multi-base metal materials such as the Cantor alloy. A certain degree of control over the NP size can be obtained by varying the laser parameters, such as fluence, wavelength, and pulse duration [11,25,30–31]. Doñate-Buendía et al. reduced the bimodality by employing a picosecond delayed double-pulse laser configuration [32]. In all the previous cases, the spatial profile of the laser beam employed was Gaussian. Here we propose to apply a novel approach of spatial beam shaping to modify the NP size distribution obtained by PLAL using a donut-shaped spatial beam profile.

Donut-shaped laser beams, with and without orbital angular momentum, have been recently demonstrated to be very efficient in surface micro- and nanostructuring [33–37] and in laser additive manufacturing [38]. They enable the formation of different structures whose size and shape can be precisely controlled. Furthermore, donut-shaped beams enable a considerable increase in the radiation absorption efficiency inside transparent materials because of the implosion of the absorbed energy during propagation [39].

It is crucial to understand the early-stage mechanisms of PLAL to correlate them to NP size control. Plasma plume formation and expansion is a critical step that occurs after absorption of the laser radiation and localized heating of the target surface. The plume interacts with the surrounding liquid. This interaction defines the cooling rate of the species present in the plasma and significantly affects NP growth (i.e., plasma quenching forms small NPs). The plasma temperature and pressure determine the cavitation bubble and NP formation [40–41].

The plasma plume that heats up the liquid causes liquid vaporization and subsequent bubble nucleation. The initial pressure of the bubble is very high (higher than 1 GPa) allowing it to expand until it reaches equilibrium with the outer pressure, achieving the bubble maximum size. The bubble then collapses, and the process can be repeated for several cycles. Ions, atoms, and clusters within the confined bubble nucleate and grow to form the NPs. The bubble collapse can induce shockwaves that break up the NP agglomerates [42–43]. The NPs are released from the cavitation bubble mainly during bubble collapse. Jet formation can penetrate the bubble boundary and expels NPs outside the bubble, or the formed shockwave can push NPs into the liquid phase.

The modification of the beam shape inherently changes the spatial distribution of the laser intensity and, thus, the radiation absorption by the target, influencing plasma plume and cavitation bubble formation, evolution, cooling, and the temperature and pressure conditions that determine nanoparticle formation. In the case of a donut-shaped beam, the plasma temperature and pressure distribution differ from the plasma in a Gaussian beam. These significant differences in pressure and temperature distribution can play a role in the formation of bubbles with different morphology and different expansion/collapsing rates. Recent simulations of plasma plumes induced by donut-shaped pulses [44] predicted unusual plume expansion dynamics with the formation of multiple internal shock waves, which strongly increase plume density and temperature. It can be expected that donut-shaped laser pulses can also offer new possibilities for NP production via PLAL by affecting plume geometry and dynamics. Thus, the NP formation mechanism and the resulting size distribution are modified compared to a Gaussian beam. However, as far as we know, donut-shaped laser pulses have not yet been investigated under PLAL conditions.

In this study, PLAL with donut-shaped beams of controlled polarization was performed to produce nanoparticles from metal (gold), oxide (yttrium oxide, Y_2_O_3_), and alloy (high-entropy alloy (HEA)) targets. The selected materials are typical representatives of their classes, whose NPs are widely used in applications such as biology and medicine for gold [9,29], strengthened ceramics and steels for high-temperature applications for Y_2_O_3_ [45–47], and catalysis and energy storage for HEAs [48–49]. The produced NPs are compared with those obtained with Gaussian beams. The evolution of the PLAL-generated cavitation bubble dynamics was investigated by using shadowgraphy method for both beam shapes.

## Materials and Methods

### Targets for PLAL

Plate targets composed of high-purity gold (AGOSI AG, Germany) and Y_2_O_3_ (Porzellanfabrik Hermsdorf GmbH, Germany) with dimensions of ca. 10 × 10 × 1 mm^3^ were used to produce gold and yttrium oxide NPs, respectively, by PLAL. The Cantor HEA CoCrFeNiMn target of equimolar composition was prepared by employing powder pressing as described in [18]. In brief, elemental powders of Co (purity 99.8%), Cr (99.2%), Fe (99.5%), Mn (99.6%), and Ni (99.9%) with a particle size less than 10 µm (Thermo Fisher Scientific, USA) were mixed, homogenized, and pressed at 200 MPa (Datona press, Netherlands) into a 2 mm thick target with 10 mm diameter. The pressed disc was then sintered in a furnace (Carbolite-CTF, UK) under vacuum conditions at 1000 °C for 22 h. Finally, the sintered disc was mechanically polished to remove any possible oxide layer from the surface.

### PLAL NP generation

**Figure 1:**
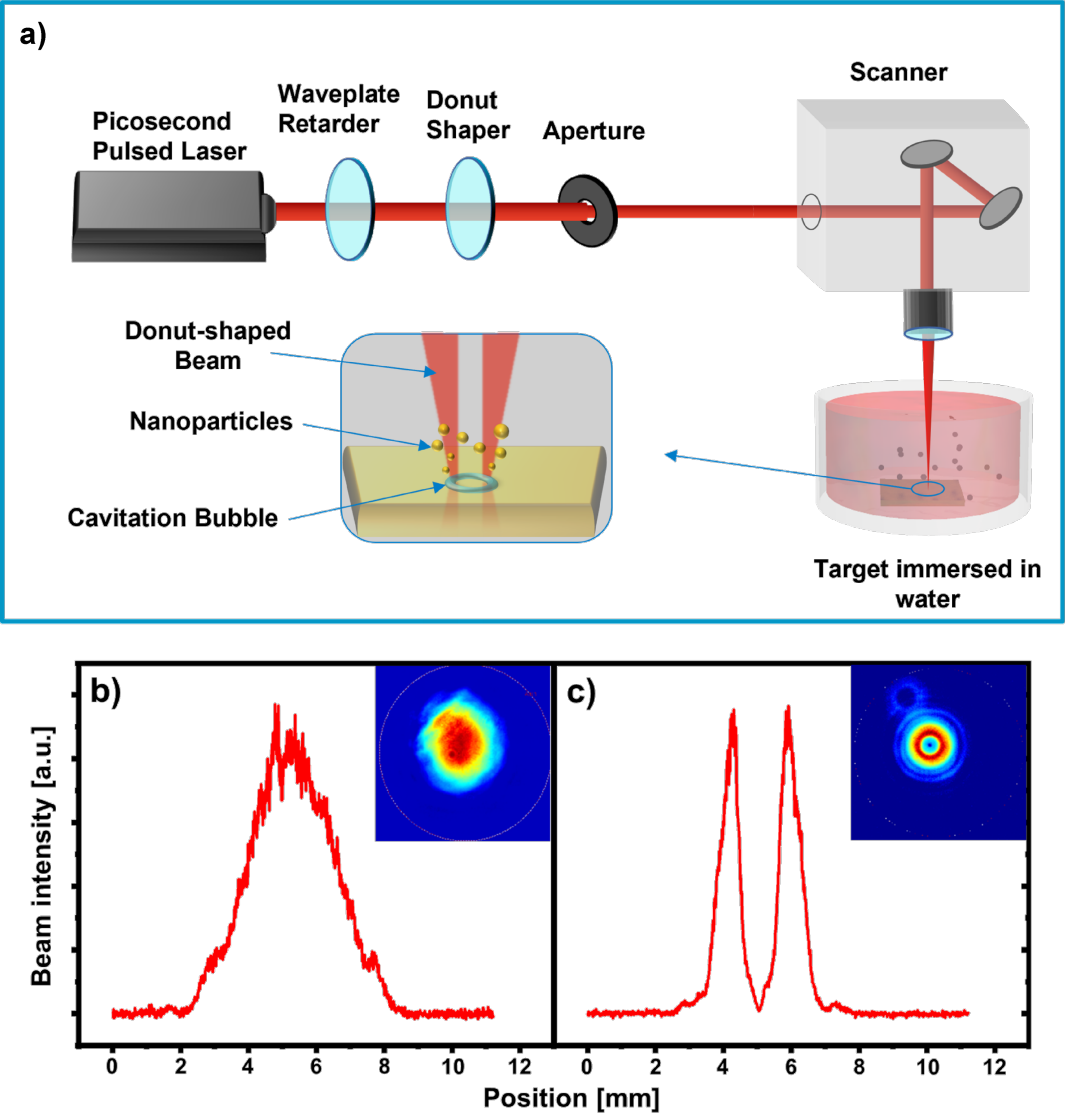
(a) Schematic of the experimental setup for beam shaping and PLAL. (b, c) Intensity profiles of the Gaussian and donut-shaped beams, respectively, derived from the profiler images (insets in (b) and (c)).

Figure 1 shows a general scheme of the experimental setup. Each ablated target was positioned in the flow-through PLAL chamber [50] with a liquid volume of ca. 5 mL and laser-irradiated at normal incidence through a glass window. The distance between the target surface and the window was ca. 7 mm. Deionized water was used as liquid in all experiments and pumped through the chamber at a flow rate of 0.13 L·min^−1^, ensuring the complete filling of the chamber volume under laminar flow conditions. For the synthesis of gold and Y_2_O_3_ NPs, PLAL was performed using a high-power picosecond laser HyperRapid NXT (Coherent Corp., USA, pulse duration 10 ps, wavelength 1064 nm, maximum power 120 W) and a Galvo-scanner SUPERSCAN IV-15 (Raylase GmbH, Germany) equipped with an f-theta lens (focal distance 167 mm). The targets were irradiated at 400 kHz repetition rate for 2 min along a spiral path with an outer diameter of 10 mm and a scanning speed of 20 m·s^−1^. The pulse energy was varied in the 50–250 µJ range by a laser attenuator to produce colloids with NP concentrations in the range of several tens of milligrams per liter. HEA NPs were synthesized using a middle-class 6 ps, 1030 nm Yb:KGW laser PHAROS (Light Conversion, Vilnius, Lithuania) and a ScanCube IV galvanometric scanner (Scanlab, Pichheim, Germany) equipped with a 163 mm focal length lens. The irradiation conditions were 50–250 µJ pulse energy range, 10 kHz pulse repetition rate, 40 min irradiation time, 2 m·s^−1^ beam scanning speed, and 4 × 4 mm^2^ ablated area with a zig-zag scan pattern.

To generate donut-shaped laser pulses from the initial Gaussian profile, an S-waveplate convertor (Workshop of Photonics, Vilnius, Lithuania) was used (Figure 1a). A half-waveplate retarder was added in front of the S-waveplate to control the polarization state of the donut-shaped pulses between radial and azimuthal. The shapes of the beams were monitored on a temporarily reflected beam branch using a profiler (typical shapes of the Gaussian and donut-shaped beams are shown in Figure 1b and Figure 1c, respectively). To monitor the polarization of the donut-shaped beam, an additional Glan–Taylor polarizer was placed in front of the profiler. The spot diameters, 2*w*_o_, of the Gaussian beams (where *w*_o_ is the beam waist) at the sample surface were measured with a silicon target using the *D*^2^ technique [51], obtaining similar spot sizes of 65 µm for the PHAROS laser and 72 µm for the NXT laser (1/*e*^2^ criterion). To evaluate the effective spot for the donut-shaped beam, we measured the pulse energy required to induce silicon damage and found that it is ca. 2.7 times higher than that for the Gaussian beam. This indicates that the peak fluence of the donut-shaped pulse is lower by a factor of *e* = 2.7 than that of the Gaussian pulse under the same focusing conditions, in agreement with simulations [36].

To investigate the cavitation bubble formed by a single pulse employing the standard Gaussian beam or the donut-shaped beam, shadowgraph images were taken using a high-speed camera (Andor iStar DH334T-18H-13, Oxford Instruments, England) equipped with a xenon flash lamp (L4633-01, Hamamatsu, Japan). A detailed description of the experimental setup can be found in a previous work [52]. The top-view setup was applied to observe the bubble morphology evolution on a transparent yttrium aluminum garnet (YAG) target. The YAG crystal has a low thermal conductivity and a high laser-ablation threshold, minimizing the influence of the ablation products on cavitation bubble dynamics and, thus, observing the pure effect of beam-shaping on the laser-induced cavitation bubble. In these experiments, the laser pulse energy was set to 400 µJ for both beams with identical focusing conditions. A telecentric telescope (Correctable T/1.5, Sill Optics, Germany) was used for magnification.

To characterize the morphology and size of the NPs synthesized by PLAL, the colloidal samples were drop-cast on a silicon wafer and dried for microscopic analysis. All NPs were characterized using scanning electron microscopy (SEM, Quanta 400 FEG, FEI Company, USA and TESCAN MIRA3 LMH, Brno, Czech Republic). The crystal structure of the HEA NPs was determined by X-ray diffraction (XRD) using a Smartlab diffractometer (Rigaku, Japan).

SEM was used to characterize the nanoparticle size distribution and to determine the beam shape influence while maintaining comparable PLAL parameters for both beams to minimize the size deviation influenced by factors such as structural and compositional variations of the NPs.

All colloidal samples are gathered directly after the PLAL process to minimize the effect of particle aggregation and isolate the laser beam shape effect on particle size.

The employment of different materials is intended to clarify the effect of the donut-shaped beam on NP size and to approve whether factors such as phase and composition can be disregarded. For example, the inertness of gold minimizes its oxidation by laser irradiation, whereas the HEA is composed of elements prone to oxidation, especially in water. However, a similar trend for the different materials using the donut-shaped PLAL can indicate that the donut-shaped beam does not drastically modify the materials’ composition. This will be discussed in section Results and Discussion.

## Results and Discussion

**Figure 2:**
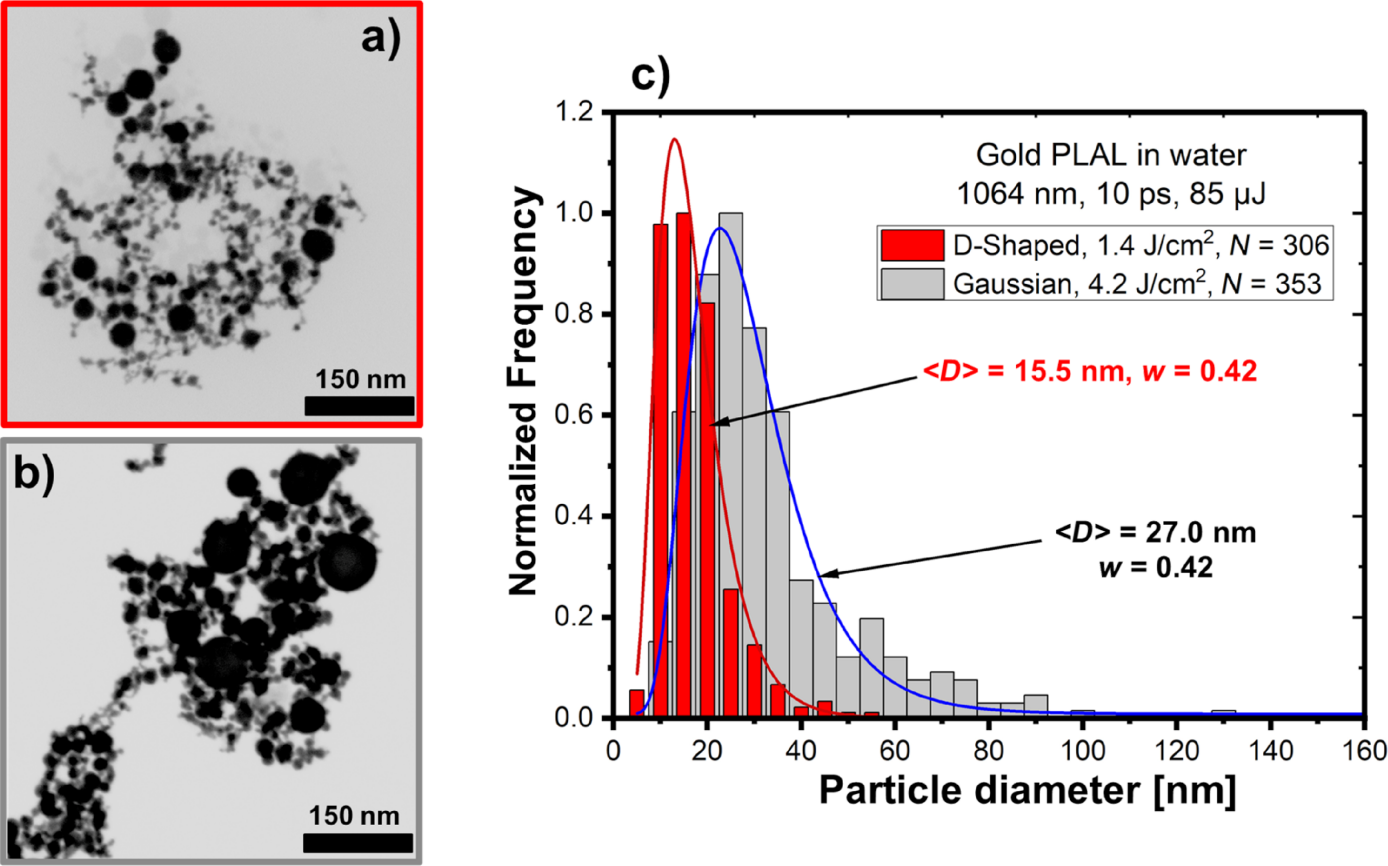
Comparison of gold NPs produced by PLAL with radially polarized donut-shaped and Gaussian laser beams at a pulse energy of 85 µJ. (a, b) SEM micrographs of NPs obtained using the donut-shaped and Gaussian beams, respectively. (c) NP size distributions showing the number of analyzed NPs (*N*), the median diameter (⟨*D*⟩), and the polydispersity index (*w*) values derived from the log-normal approximations (solid lines).

Figure 2 depicts the size distributions of the gold NPs produced by PLAL using donut-shaped and Gaussian beams at a relatively low pulse energy of 85 µJ for both beams (fluences are 1.4 and 4.2 J·cm^−2^, respectively). The fraction of small NPs with enhanced size uniformity is higher when using the donut-shaped beam, and NPs larger than 50 nm are barely detectable (Figure 2a). In contrast, for the Gaussian beam, the distribution extends up to 100 nm (Figure 2b). Figure 2c shows the shift of the median value ⟨*D*⟩ from 27.0 nm (Gaussian beam) to 15.5 nm (donut-shaped beam) while maintaining the same value of the polydispersity index *w*.

An increase in the pulse energy of the donut-shaped beam by a factor of around 2.6 (i.e., 217 µJ) to achieve nearly the same fluence as for the Gaussian beam at 85 µJ (Figure 2c) does not significantly affect the NP size distribution, (Figure S1, Supporting Information File 1).

In addition, the effect of the pulse energy of the radially polarized donut-shaped beam on the size of the gold NPs was investigated. When using different pulse energies, 85 and 217 µJ, no significant variation in the gold NP size distribution was observed (Figure S1, Supporting Information File 1). Furthermore, it was found that the polarization of the donut-shaped beam (radial or azimuthal) barely influences the size distribution of the gold NPs. Similarly, the sizes of other studied PLAL-produced NPs also exhibited weak dependencies on the polarization of the donut-shaped beam (Figure S2, Supporting Information File 1). Therefore, results obtained only with the radially polarized donut-shaped beam will be presented below.

**Figure 3:**
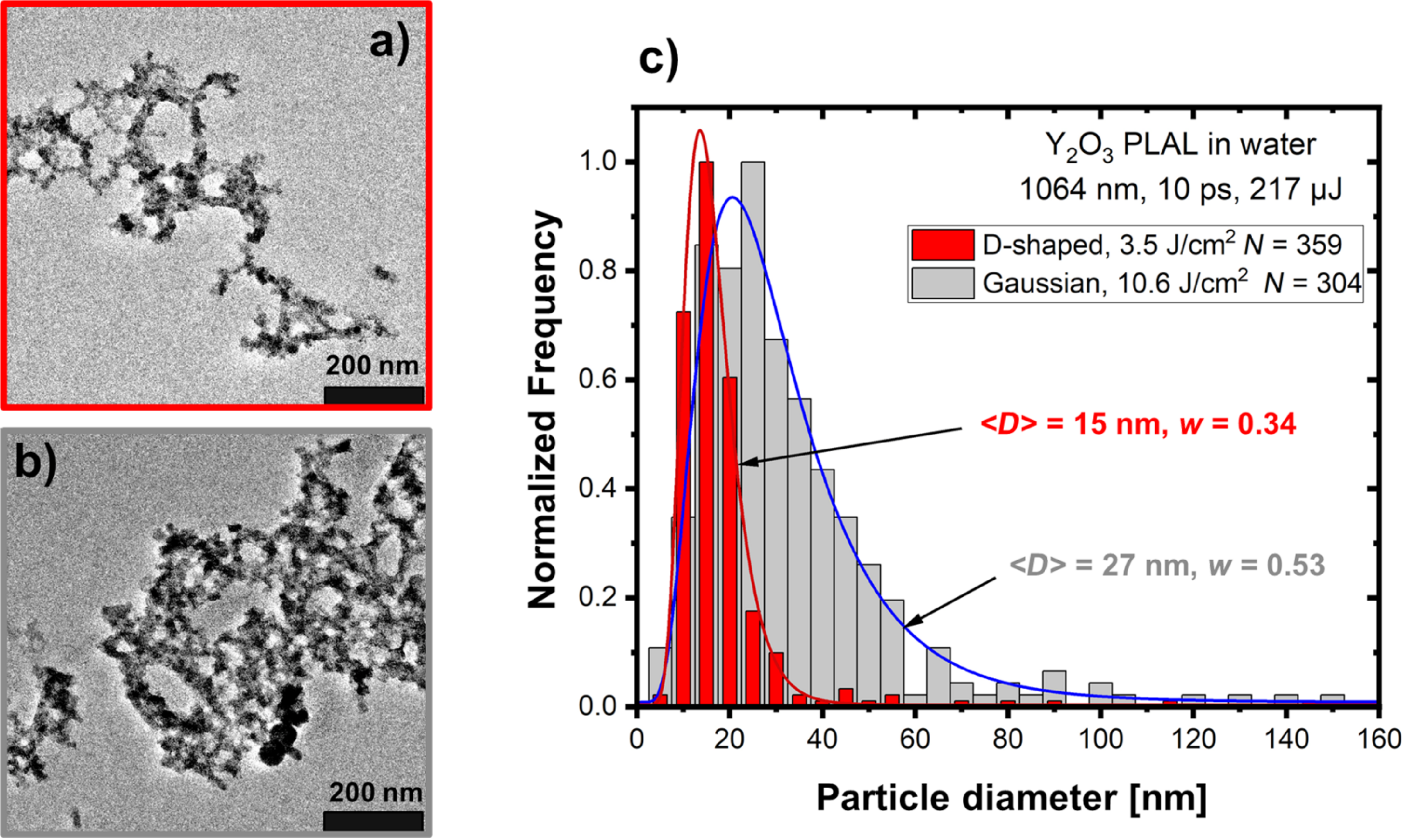
Comparison of Y_2_O_3_ NPs produced by PLAL with donut-shaped and Gaussian laser beams at a pulse energy of 217 µJ. (a, b) SEM micrographs of NPs obtained using donut-shaped and Gaussian beams, respectively. (c) NP size distributions showing the number of analyzed NPs (*N*), the median diameter (⟨*D*⟩), and the polydispersity index (*w*) values derived from the log-normal approximations (solid lines).

Figure 3 compares SEM images and size distributions of Y_2_O_3_ NPs produced by PLAL with the donut-shaped and the Gaussian beams at a pulse energy of 217 µJ for both beams (fluences of 3.5 and 10.6 J·cm^−2^, respectively). For the donut-shaped pulses (Figure 3a), the produced NPs are smaller than for the Gaussian pulses (Figure 3b) with a similar decrease in the mean NP size from 27 nm (Gaussian beam) to 15 nm (donut-shaped beam) as observed for gold NPs in Figure 2. Similar to the gold NPs, there is a noticeable difference in the cut-off of the distribution obtained with the donut beam, ⟨*D*⟩ ≈ 40 nm, compared to the Gaussian pulses where there is a pronounced tail in the particle size distribution extending beyond 100 nm. Besides, the distribution is considerably narrower for the donut-shaped beam, *w* = 0.34, than for the Gaussian beam, *w* = 0.53. Furthermore, PLAL with donut-shaped pulses improves the shape of Y_2_O_3_ NPs, which are more regular (Figure 3a) than the rather elongated and aggregated NPs produced with the Gaussian pulses (Figure 3b).

**Figure 4:**
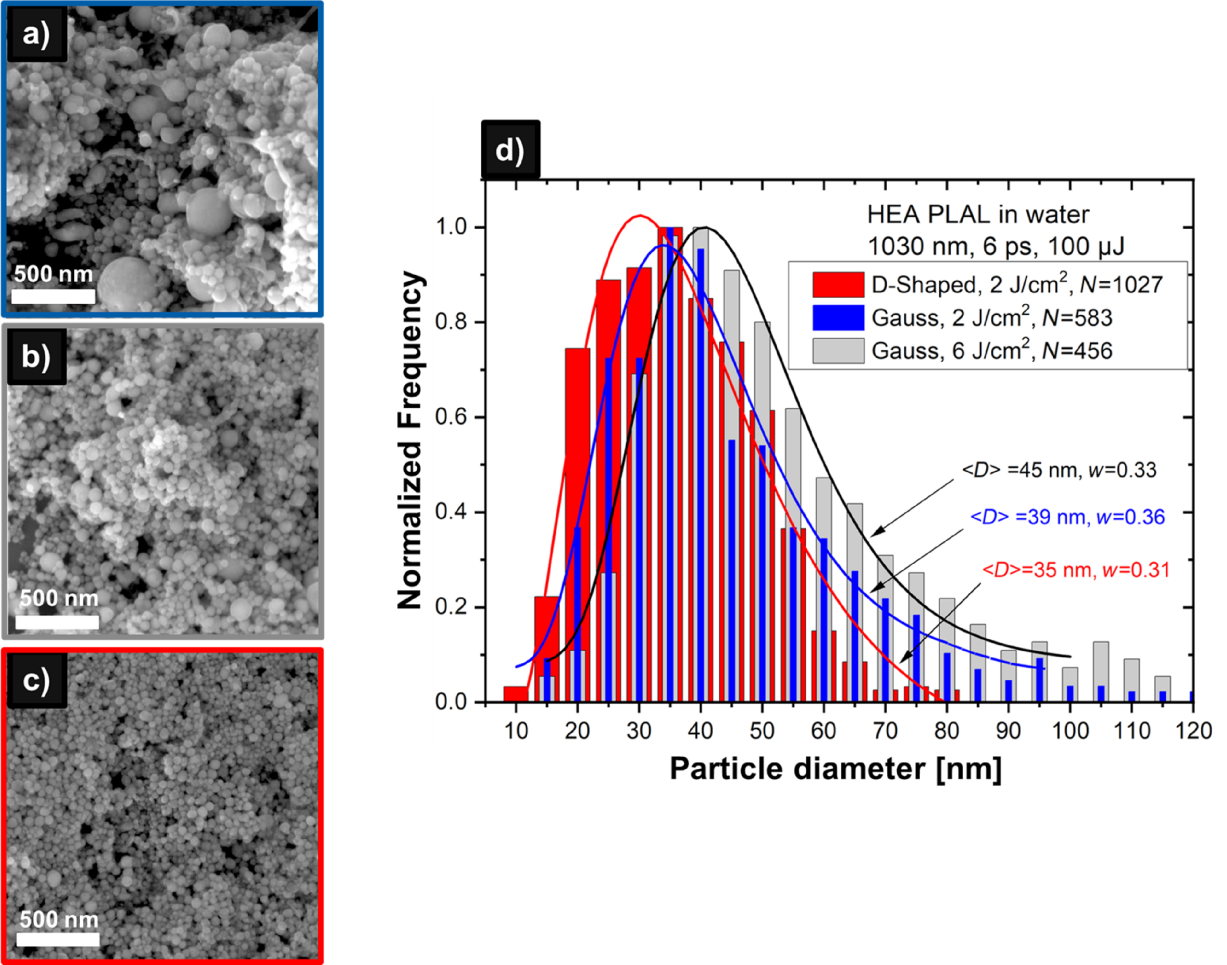
Comparison of HEA NPs produced by PLAL with Gaussian and donut-shaped laser beams at a pulse energy of 100 µJ. (a, b) SEM micrographs of NPs obtained with the Gaussian beam at fluences of 6 and 2 J·cm^−2^, respectively. (c) SEM image of NPs obtained with the donut-shaped beam at a fluence of 2 J·cm^−2^. (d) NP size distributions showing the number of analyzed NPs (*N*), the median diameter (⟨*D*⟩), and the polydispersity index (*w*) values derived from the log-normal approximations (solid lines).

Figure 4c shows an SEM image and the size distribution of HEA NPs obtained with donut-shaped beams (100 µJ pulse energy, 2 J·cm^−2^ fluence) in comparison with two PLAL regimes of the Gaussian laser beams, namely, at the same pulse energy and focusing conditions (i.e., at a fluence of 6 J·cm^−2^ (Figure 4a)) and at the same laser fluence of 2 J·cm^−2^ (Figure 4b). In the latter case, the HEA target was shifted from the focal position to increase the irradiation spot area about threefold. The NPs obtained with the donut-shaped beam with their average size ⟨*D*⟩ = 35 nm are smaller, and their distribution is narrower than those of the NPs synthesized in both regimes of Gaussian pulses, ⟨*D*⟩ = 45 and 39 nm. In addition, similar to gold and yttrium oxide, the NPs produced with donut-shaped pulses are more regular in shape with a reduced amount of large and aggregated particles (Figure 4c). This results in a cut-off of the size distribution at around 80 nm in contrast to the Gaussian beams, where both distributions exhibit high-size tails extending well beyond 100 nm, especially for the high-fluence regime. A two-fold increase of the pulse energy of the donut-shaped pulses, from 100 to 200 µJ, leads, in this case, to an increase of the average size of the HEA NPs from 35 to 55 nm (Figure S3, Supporting Information File 1). Again, the produced NPs exhibit regular spherical shapes with a relatively narrow size distribution, and almost no aggregated particles are observed.

The XRD analysis indicates that the obtained HEA NPs are partially oxidized and have a relatively low degree of crystallinity compared to the original target (Figure S4, Supporting Information File 1). The crystalline to amorphous transition is likely due to the development of stresses induced by ultrafast heating and cooling during PLAL [53]. However, both oxidation and phase transformation processes do not seem to have a major impact on the resulting sizes of NPs produced with donut-shaped pulses since the observed trends of size reduction and narrowing the distribution are similar to those for the other studied materials. The peak corresponding to the crystallographic plane (111) is very small but visible, whereas the other (200) and (220) peaks are not visible for the NPs. Laser irradiation plays a major role in the formation of HEA with strong (111) peak while minimizing (200) and (220) peaks [54].

The observed NP size-reduction trend for the donut-shaped laser pulses was observed for all studied materials and in every of the three repeated PLAL experiments performed for each material at a fixed pulse energy, including the experiments with different beam polarizations (radial and azimuthal). To ensure the reproducibility of the results, a minimum of 300 NPs per characterized sample were analyzed for every single experiment performed.

The tendencies can be explained by the fact that a donut-shaped beam avoids overheating in the center of the laser spot and prevents underheating in the peripheral region with temperatures below the ablation threshold. In contrast, ablation with a Gaussian beam results in the ejection of molten droplets from the overheated central region and the formation of a crater rim. For instance, it has been demonstrated that during vortex-beam ultrashort laser ablation of thin metal films [55] and tungsten targets in air [56], the ablation crater reproduces the beam intensity distribution at low laser fluence. At high fluence, the crater exhibits a uniform shape without chaotic hydrodynamically driven relief. According to Shih et al. [23,57], the mechanism of NP formation and the generation of large NPs is related to the hydrodynamic instability of the decelerating plume interpenetrating the liquid’s low-density region, the metal vapor condensation, and the emission of metal nanojets. Thus, the donut-shaped beam, which forms a more homogeneous temperature field than the Gaussian beam, affects the material melt’s hydrodynamics over the laser spot area and leads to the formation of smaller NPs with a narrower size distribution.

**Figure 5:**
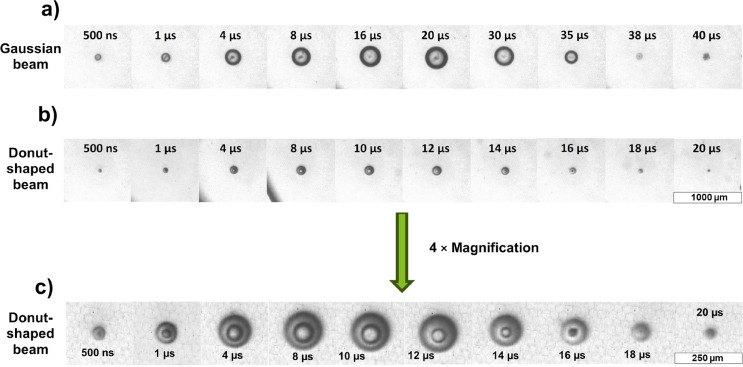
Shadowgraph images of the cavitation bubbles produced by (a) Gaussian pulse and (b) donut-shaped pulse at a pulse energy of 400 µJ for both beams at different moments in time. (c) Images in (b) at a higher magnification.

Therefore, the experiments with different materials demonstrate that PLAL with donut-shaped laser pulses enabled efficient control of NP synthesis, achieving smaller average sizes, narrower distributions, and higher sphericity. The changes in particle sizes may also be attributed to distinct cavitation bubble dynamics induced by the donut-shaped beam (Figure 5).

While a detailed investigation into cavitation dynamics is beyond the scope of this paper, preliminary observations suggest that the unique structure and shorter lifetime of cavitation bubbles generated by donut-shaped pulses may contribute to the enhanced nanoparticle size control. Reich et al. studied the effect of cylindrical defocusing on PLAL cavitation bubble shape and dynamics [58]. At the same pulse energy (but five times lower fluence), the line-shaped ablation developed bubble sphericity from a value less than unity (flat, elongated bubble) towards that of a spherical bubble. In contrast, the round focus caused a longer-living, hemispherical bubble almost from the start, and the bubble rebound behavior was completely different. The maximum size (and thereby the lifetime) of the bubble was significantly reduced for the donut-shaped beam compared to the tight focus, which was attributed to the altered bubble shape. The donut beam shape appears to influence the bubble dynamics, potentially creating a different environment for particle formation and reducing the particle growth and agglomeration tendencies. The cavitation bubble created by the donut beam forms a toroidal structure that overruns the ring-shaped ablation site, temporally leaving that spot uncovered (Figure 5b,c), unlike the quasi-hemispherical bubble produced by the Gaussian beam, which covered the ablation spot until collapse (Figure 5a). Such toroidal bubbles are well known for ring-shaped laser focusing into free liquids [59–60], and toroidal shapes have also been reported for symmetrical bubble collapse near walls [61–62]. But here, we investigate the laser ablation of a solid target, not the liquid, and link the ablation beam’s donut shape to NP quality. Future studies may examine how these modified plume shapes, plume dynamics, and bubble lifetimes impact nanoparticle synthesis, particularly concerning their size distribution and morphological uniformity.

## Conclusion

Nanoparticles of three materials, namely, gold, yttrium oxide, and high-entropy alloy, were synthesized by pulsed laser ablation in water using picosecond donut-shaped laser pulses and compared with NPs produced by conventional Gaussian pulses. With all materials, it is demonstrated that donut-shaped pulses provide significant advantages regarding PLAL synthesis, yielding smaller NPs with narrower size distributions and more regular shapes.

The observed effects in the NP synthesis are likely due to a more uniform heating of the ablation spot by a donut-shaped pulse, affecting the hydrodynamics of the melt within the spot. Preliminary observations suggest that the distinct cavitation bubble dynamics produced by donut-shaped pulses may also play a role in preventing nanoparticle growth and agglomeration. These results indicate that donut-shaped laser pulses offer a promising approach for more precise control over nanoparticle size and shape uniformity, though further studies are needed to fully elucidate the mechanisms involved.

## Supporting Information

File 1 Additional experimental data.

## Data Availability

Data generated and analyzed during this study is available from the corresponding authors upon reasonable request.
