# Supplementary material for: Size control of nanoparticles synthesized by pulsed laser ablation in liquids using donut-shaped beams
Source: Beilstein J Nanotechnol. 2025 Mar 25;16:407–17. doi: 10.3762/bjnano.16.31 (PMC11956078; doi:10.3762/bjnano.16.31)
Supplement: File 1 — Additional experimental data. [file Beilstein_J_Nanotechnol-16-407-s001.pdf]

BEILSTEIN JOURNAL OF NANOTECHNOLOGY

Supporting Information

for

Size control of nanoparticles synthesized by pulsed laser ablation in liquids using donut-shaped beams

Abdel Rahman Altakroury, Oleksandr Gatsa, Farbod Riahi, Zongwen Fu, Miroslava Flimelová, Andrei Samokhvalov, Stephan Barcikowski, Carlos Doñate-Buendía, Alexander V. Bulgakov and Bilal Gökce

Beilstein J. Nanotechnol. **2025,** *16,* 407–417. doi:10.3762/bjnano.16.31

Additional experimental data

License and Terms: This is a supporting information file under the terms of the Creative Commons Attribution License (https://creativecommons.org/licenses/by/4.0). Please note that the reuse, redistribution and reproduction in particular requires that the author(s) and source are credited and that individual graphics may be subject to special legal provisions.
The license is subject to the *Beilstein Journal of Nanotechnology* terms and conditions: (https://www.beilstein-journals.org/bjnano/terms)

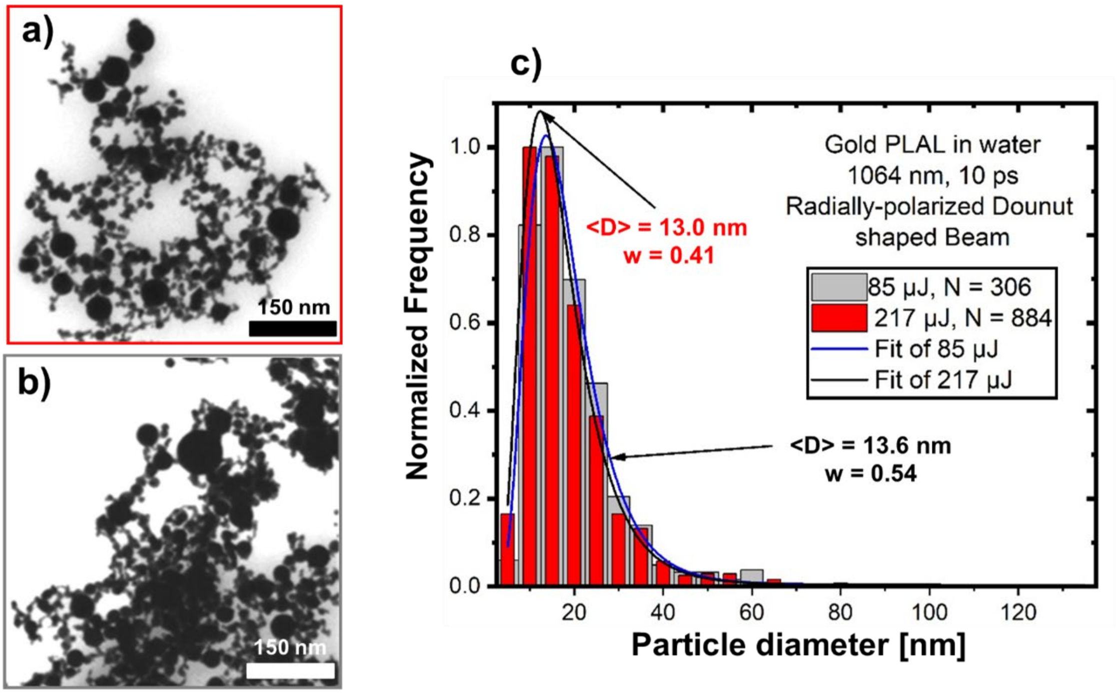

Figure S1: Comparison of gold NPs produced by PLAL with radially polarized donut-shaped laser beams at pulse energies of 85 and 217 µJ. (a, b) SEM micrographs of NPs obtained using pulse energies of 85 and 217 µJ, respectively. (c) NP size distributions showing the number of analyzed NPs (N), the median diameter ($\langle D \rangle$), and the polydispersity index (w) values derived from the log-normal approximations (solid lines).

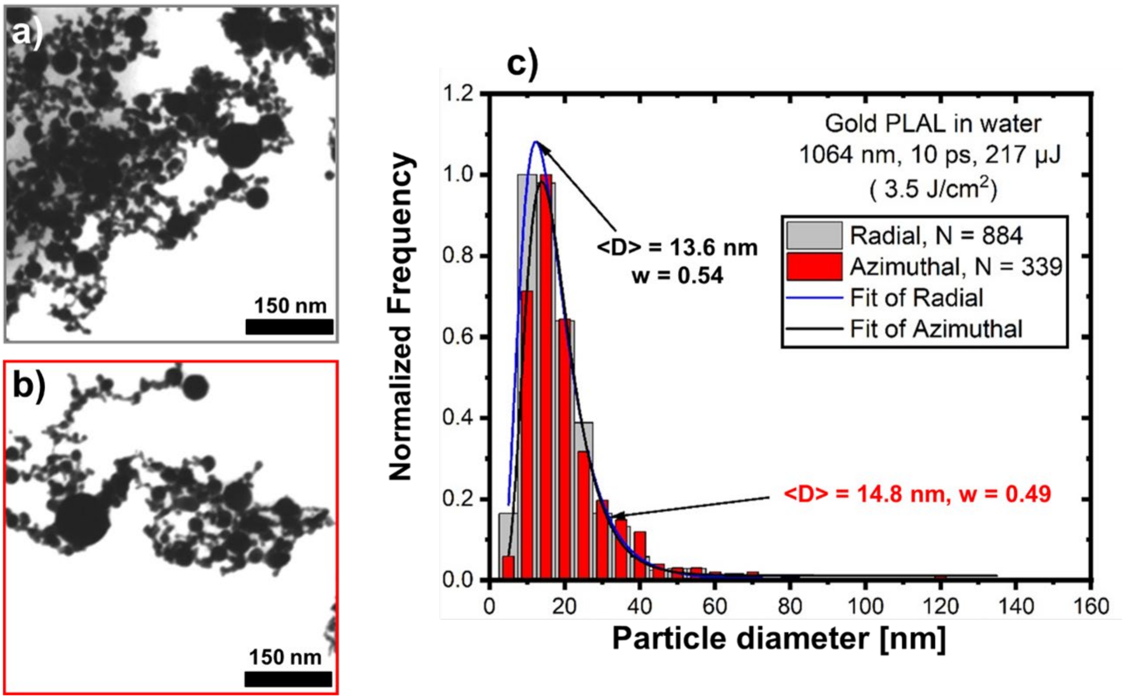

Figure S2: Comparison of gold NPs produced by PLAL with radially polarized donut-shaped and azimuthally polarized donut-shaped laser beams at a pulse energy of 217 μJ. (a, b) SEM micrographs of NPs obtained using the radially polarized donut-shaped and azimuthally polarized donut-shaped beams, respectively. (c) NP size distributions showing the number of analyzed NPs (*N*), the median diameter (⟨*D*⟩), and the polydispersity index (*w*) values derived from the log-normal approximations (solid lines).

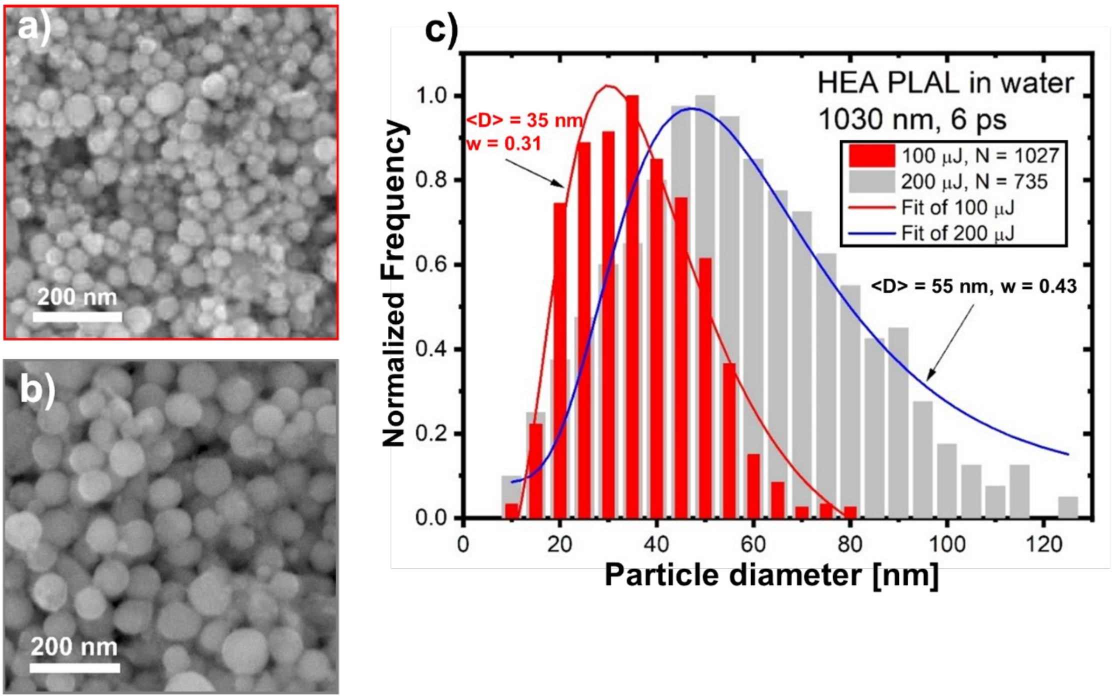

Figure S3: Comparison of HEA NPs produced by PLAL with radially polarized donut-shaped laser beams at pulse energies of 100 and 200 μJ. (a, b) SEM micrographs of NPs obtained using pulse energies of 100 and 200 μJ, respectively. (c) NP size distributions showing the number of analyzed NPs (N), the median diameter ($\langle D \rangle$), and the polydispersity index (w) values derived from the log-normal approximations (solid lines).

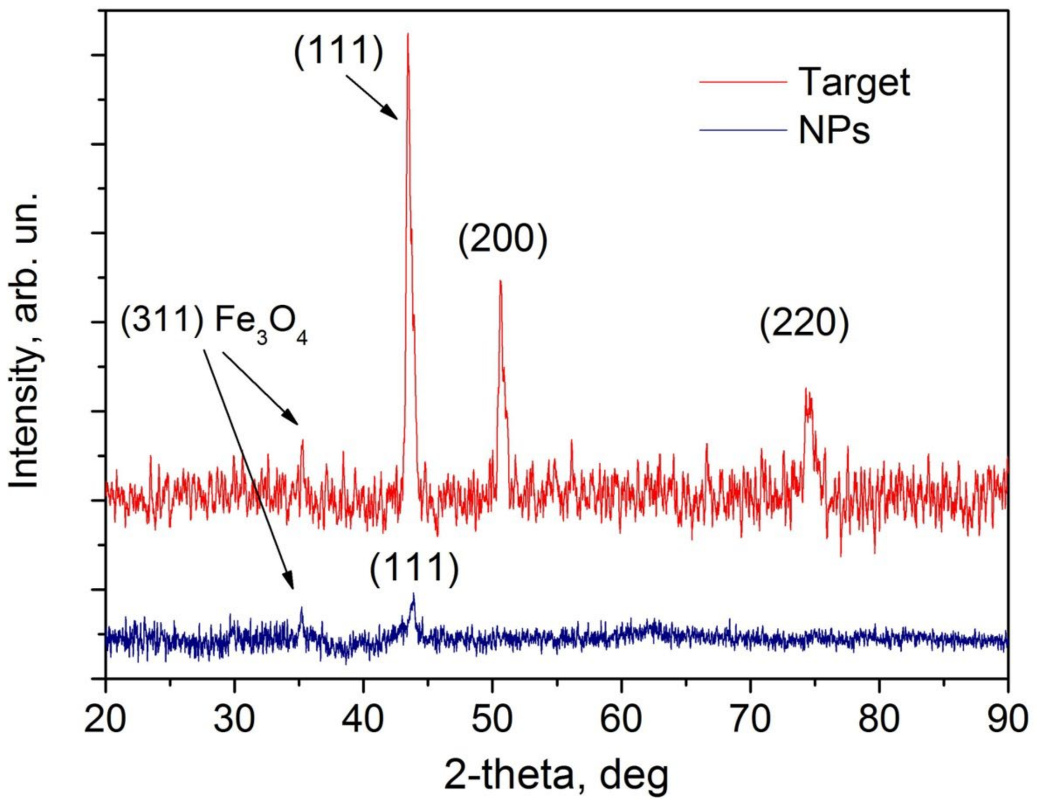

Figure S4: XRD patterns for the HEA target and NPs generated by PLAL by a donut-shaped laser beam at a pulse energy of 100 μJ. The crystalline HEA phase is represented by peaks corresponding to (111), (200), and (220) Bragg reflections of the face-centered cubic lattice. An additional (311) peak at 2θ = 35.2° corresponds to the cubic Fe_3O_4 phase. The height ratio of the oxide peak to the (111) peak is higher for NPs.
